# Optical coherence tomography in a patient with chloroquine-induced maculopathy

**DOI:** 10.4103/0301-4738.53071

**Published:** 2009

**Authors:** Suresh Ramchandani, Sushama Ramchandani

**Affiliations:** Shivam Eye Clinic and Surgical Centre, Snehal Co-Op Housing Society, Plot No 29, Sector 17, Nerul, Navi Mumbai - 400 706, Maharashtra, India

Dear Editor,

We read with interest the article ‘Optical coherence tomography (OCT) in a patient with chloroquine- induced maculopathy’ by Korah *et al*.[1]

We must congratulate the authors for the excellent article but we would like to make a few points.

1. Why was the patient started on chloroquine rather that hydroxychloroquine? It is well-documented that hydroxychloroquine has a lower incidence of eye complications compared to chloroquine.[2]
2. Was the patient regular about her follow-up schedules? What were the findings in the examination done just before the complications were noted? Did the patient have any corneal deposits? Corneal deposits though innocuous are the most commonly described ophthalmological findings following chloroquine therapy.[2] Was a visual field examination done at that time? Were there any retinal pigment epithelium (RPE) alterations? What was the protocol for taking fundus pictures and /or visual field examinations?
3. Examination and perimetry reliably showed chloroquine toxicity and yet the patient was not told to stop chloroquine but the drug was changed to hydroxychloroquine. Hydroxychloroquine was stopped and methrotrexate started after four months when the patient complained of subjective symptoms. In our opinion chloroquine/hydroxychloroquine should have been stopped earlier.
4. Any proposed screening test needs to detect retinal lesions at a stage where intervention can reverse the condition or prevent deterioration. If the cases of retinopathy detected by monitoring fail to respond to cessation of therapy, the monitoring does not have a useful role.[3]
  In this case the optical coherence tomography (OCT) findings of significant thinning were seen in a patient who had well-documented changes on fundus examination and perimetry examination. It would be interesting to know if the thinning occurs before any other change can be noted on regular examination. If not, OCT has no role whatsoever.
5. Multifocal ERG (mfERG) is a very sensitive test for detection of early retinal abnormalities under chloroquine/hydroxychloroquine therapy. Multifocal ERG can reliably detect retinal functional loss associated with chloroquine/hydroxychloroquine retinopathy. In some patients the mfERG showed reduced response amplitudes when other functional tests or morphologic examinations were conducted. In addition, follow-up studies demonstrated a decline of retinal function when using hydroxychloroquine and improvement of retinal function after discontinuation of treatment.[4] In our opinion, an mfERG would diagnose hydroxychloroquine retinopathy at the earliest stage and should be done whenever possible.
